# Oral Immunogenicity in Mice and Sows of Enterotoxigenic Escherichia Coli Outer-Membrane Vesicles Incorporated into Zein-Based Nanoparticles

**DOI:** 10.3390/vaccines8010011

**Published:** 2019-12-31

**Authors:** Jose Matías, Ana Brotons, Santiago Cenoz, Isidoro Pérez, Muthanna Abdulkarim, Mark Gumbleton, Juan M. Irache, Carlos Gamazo

**Affiliations:** 1 Department of Microbiology and Parasitology, Institute of Tropical Health, University of Navarra, 31008 Pamplona, Spain; jmatias@alumni.unav.es; 2 Department of Pharmacy and Pharmaceutical Technology, University of Navarra, 31008 Pamplona, Spain; abrotons@alumni.unav.es (A.B.); jmirache@unav.es (J.M.I.); 3 Blue Agro Bioscience, Parque Tecnológico Miramón. Paseo Mikeletegi, 54. 20009 San Sebastián, Spain; santiago.cenoz@dfblueagro.com; 4 Agropecuario Obanos, Marcilla, 31340 Navarra, Spain; isidoro@grupoobanos.com; 5 School of Pharmacy and Pharmaceutical Sciences, Cardiff University, Cardiff CF10 3NB UK; AbdulkarimMF@cardiff.ac.uk (M.A.); gumbleton@cardiff.ac.uk (M.G.)

**Keywords:** vaccine, outer membrane vesicles, ETEC, Escherichia coli, nanoparticles

## Abstract

Enterotoxigenic Escherichia coli (ETEC) strains are a major cause of illness and death in neonatal and recently weaned pigs. The immune protection of the piglets derives from maternal colostrum, since this species does not receive maternal antibodies through the placenta. In the present study, outer membrane vesicles (OMVs) obtained from main ETEC strains involved in piglet infection (F4 and F18 serotypes), encapsulated into zein nanoparticles coated with Gantrez^®®^ AN-mannosamine conjugate, were used to orally immunize mice and pregnant sows. Loaded nanoparticles were homogeneous and spherical in a shape, with a size of 220–280 nm. The diffusion of nanoparticles through porcine intestinal mucus barrier was assessed by a Multiple Particle Tracking technique, showing that these particles were able to diffuse efficiently (1.3% diffusion coefficient), validating their oral use. BALB/c mice were either orally immunized with free OMVs or encapsulated into nanoparticles (100 µg OMVs/mouse). Results indicated that a single dose of loaded nanoparticles was able to elicit higher levels of serum specific IgG1, IgG2a and IgA, as well as intestinal IgA, with respect to the free antigens. In addition, nanoparticles induced an increase in levels of IL-2, IL-4 and IFN-γ with respect to the administration of free OMVs. Orally immunized pregnant sows with the same formulation elicited colostrum-, serum- (IgG, IgA or IgM) and fecal- (IgA) specific antibodies and, what is most relevant, offspring suckling piglets presented specific IgG in serum. Further studies are needed to determine the infection protective capacity of this new oral subunit vaccine

## 1. Introduction

Intestinal infections are one of the most important problems in swine husbandry. Those caused by enterotoxigenic Escherichia coli (ETEC) produce particularly significant economic losses, due to its high rate of mortality, reduced weight gain and the cost of medication [1]. These infections commonly occur after birth or after weaning [2]. The losses produced by ETEC in farms can be minimized with the use of prophylactic antibiotics [1]. However, most countries have rationally restricted the use of prophylactic antibiotics in order to avoid the selection of resistant strains [3]. Thus, vaccination is the best choice to reduce the use of antibiotics in pig farming, and likely the most effective approach to control pathogenic bacterial infections [4]. Adequate vaccination of the sows before farrowing might protect piglets by passive immunization through colostrum and milk. In fact, E. coli vaccines have been used for many years to stimulate mucosal immunity of the sows and, hence, to elicit specific IgG and IgA antibodies in colostrum, conferring protection to the neonatal piglets [5,6,7]. However, the available parenteral vaccines to prevent postweaning diarrhea tend to stimulate the systemic rather than mucosal immune system [8,9,10,11,12,13]. In contrast to parenteral vaccines, mucosal vaccines are safer and induce immunity both systemically as well as in mucosal tissues. However, the special tendency of the mucosal immune system to generate tolerance is a big challenge to the development of oral vaccines [14].

The genetic plasticity of ETEC strains, their many different virulence factors, and the limited knowledge of the immunological mechanisms involved in protection makes the development of an effective vaccine against ETEC a real challenge [15,16]. Consequently, this antigenic complexity requires the use of multiple epitopes in order to protect against ETEC, with the aim of inhibiting bacterial adherence to receptors on the intestinal cells and neutralizing enterotoxins [11].

Swine ETEC strain’s pathogenicity is based primarily on two types of virulence factor: adhesins and exotoxins. In fact, the ETEC strain can be classified according to the fimbrial adhesins as F4 (K88), F5 (K99), F6 (987P), F18 and F41. ETEC strains expressing F4 or F18 fimbriae are the most commonly associated with diarrhea in weaned pigs. The presence of F4 or f18 fimbrial adhesins has been found to be significantly correlated with pathogenicity, but there are also significant differences in host response after F4 or F18 infection, for instance, different patterns in the elicited immune response, the lapse and intensity of the symptoms (i.e., diarrhea of piglets) or the timing in the intermittent bacterial shedding have been described [17]. In this study, we propose the use of outer membrane vesicles (OMVs) from the F4 and F18 serotypes, the main ETEC strains involved in piglet infection, as the source of relevant antigens. OMVs are naturally released from the outer membrane of Gram negative bacteria, including ETEC strains, during in vitro culture and during infection [18]. Analyses of ETEC OMVs components have confirmed the presence of a wide variety of main virulence factors, including lipopolysaccharides (LPS), toxins, adhesins such as flagellin (FliC), or the major molecular structural subunit of fimbriae [19]. However, the harsh conditions of the gut hamper the possibility of developing a vaccine against ETEC based on the use of free OMVs. Therefore, in this work, acid resistant and mucus permeating nanoparticles were selected as oral delivery systems for the OMVs. These nanoparticles would facilitate the arrival of the encapsulated antigens to the surface of the intestinal epithelium. The selected nanoparticles are based in zein (storage protein from corn with a GRAS status) that are coated with a hydrophilic corona made from a polymer conjugate, obtained by the covalent linkage of mannosamine to a poly(anhydride) (Gantrez^®®^ AN) backbone. The conjugate was selected for a double objective: to confer mucus-permeating properties to the nanoparticles, and to improve the immunostimulatory properties of the nanoparticles. In fact, a Gantrez^®®^ AN-based device is expected to act as an active Th1 adjuvant through TLR exploitation [20]. In addition, the presence of mannosamine could facilitate the targeting properties of these nanocarriers for dendritic cells [21]. Here, we report for the first time the immunoadjuvant capacity of this nanoparticle formulation for oral vaccination in BALB/c mice and sows.

## 2. Materials and Methods

### 2.1. Chemicals

Gantrez^®®^ AN 119 was supplied by Ashland Inc. (Barcelona, Spain). Mannosamine hydrochloride, zein, mannitol, lysine, tween 20, bromoethylamine-hydrobromide and Bovine Serum Albumin (BSA) were purchased from Sigma-Aldrich (Spain). Acetone was obtained from VWR-Prolabo (Spain) and O- phtalaldehide was provided by Invitrogen (Thermo Fisher, Waltham, Ma. USA). Ethanol, formaldehyde, NaOH and DMSO was supplied by Panreac (Spain). TSB was obtained from bioMérieux (Marcy l’Etoile, France). RPMI was obtained from Gibco-BRL (UK). Coomassie brilliant blue and sample buffer was purchased from Bio-Rad (Spain). All other reagents and chemicals used were of analytical grade.

### 2.2. Isolation of the OMVs

The *E. coli* F4 and F18 serotypes used in this study were obtained from CECT (Valencia, Spain) and Agropecuaria Obanos (Navarra, Spain), respectively. Strains were cultured in Tryptone–Soya–Broth for 18 h at 37 °C with agitation. OMVs were obtained following a method adapted from Camacho et al. [22]. Bacteria were grown in 500 mL of TSB under shaking overnight to early stationary phase (37 °C, 125 rpm). Then, bacteria were inactivated during 6 h with a solution of binary ethylenimine and formaldehyde (6 mM BEI—0.06% FA, 6 h, 37 °C). Cells were discarded by centrifugation (10,000× *g*, 10 min) and the supernatant filtered through a 0.45 µm Durapore PVDF filter (Millipore) and purified by tangential filtration using a 300 kDa concentration unit (Millipore). The retenate was frozen and subsequently lyophilized.

### 2.3. Preparation of OMV-Loaded Nanoparticles (OMV–GM–NPZ)

The nanoparticles were prepared in a two-step process [23]. In the first step, the conjugate between Gantrez^®®^ AN and mannosamine (GM) was synthetized. For this purpose, 1 g Gantrez^®®^ AN [poly(anhydride)] was dissolved in 120 mL acetone. Then, 50 mg mannosamine was added and the mixture was heated at 50 °C, under magnetic agitation at 400 rpm, for 3 h. Then, the mixture was filtered through a pleated filter paper and the organic solvent was eliminated under reduced pressure in a Büchi R-144 apparatus (BÜCHI Labortechnik AG, Switzerland) until the conjugate was totally dried. Finally, the resulting powder was stored at room temperature in a hermetically sealed container until use. The GM conjugate was characterized and the mannosamine content was calculated to be 21 µg/mg polymer.

In the second step, the OMVs-loaded zein nanoparticles prepared were coated by simple incubation with the GM conjugate. A ratio of 400 mg zein, 15 mg OMVs and 66.7 mg L-lysine were dissolved in 40 mL ethanol 70%. Then, nanoparticles were obtained by the addition of 40 mL water in the ethanol 70 % phase solution of zein. The resulting nanoparticles were maintained under magnetic agitation for 5 min. After that, 1 mL of a solution of GM in water (10 mg/ Ml) was added to the suspension of OMV-loaded zein nanoparticles and incubated for 30 min. Finally, 13.33 mL of an aqueous solution of mannitol (200 mg mannitol per 100 mg zein) was added to the mixture of nanoparticles and the mixture was dried in a Büchi R- 144 spray-drier (BÜCHI Labortechnik AG, Flawil, Switzerland). For this purpose, the following parameters were selected: inlet temperature of 90 °C, outlet temperature of 60 °C, spray-flow of 600 L/h and aspirator at 100% of the maximum capacity. The resulting nanoparticles were identified as F4-GM-NPZ (for OMV-F4 loaded nanoparticles) and F18-GM-NPZ (when OMV-F18 was encapsulated). Empty nanoparticles (GM-NPZ) were prepared in the same way as described above, but in the absence of GM.

## 3. Characterization of Nanoparticles

### 3.1. Particle Size, Zeta Potential and Yield

The particle size, polydispersity index (PDI) and zeta-potential were determined by photon correlation sprectroscopy (PCS) and electrophoretic laser Doppler anemometry, respectively, using a Zetasizer analyser system (Malvern^®®^ Instruments, UK). The diameter of the nanoparticles was determined after dispersion in ultrapure water (1/10) and measured at 25 °C by dynamic light scattering angle of 90°. The zeta potential was determined as follows: 200 μL of the samples was diluted in 2 mL of a 0.1 mM KCl solution adjusted to pH 7.4. The yield of the preparative process of nanoparticles was calculated by gravimetry.

### 3.2. Morphology and Shape

The shape and morphology of nanoparticles were examined by transmission electron microscopy. OMVs samples dispersed in ddH2O were laid on copper grids with a film of formvar (EMS, FF200-cu) for 30 s at 37 °C. The samples were washed three times with ddH2O, and finally a negative staining was performed with 3% of uranyl acetate. To visualize the vesicles, a Zeiss Libra 120 Transmission Electron Microscope (Oberkochen, Germany) coupled with a digital imaging system was employed.

### 3.3. In Vitro Evaluation of Nanoparticles Diffusion in Mucus

The diffusion of nanoparticles through porcine intestinal mucus barrier, as an in vitro measurement of their mucus-permeating properties, was assessed by Multiple Particle Tracking (MPT) technique. MPT involves the video microscopy and post-acquisition analysis of hundreds of individual particles and their trajectories within a mucus matrix [23]. In brief, 0.05 µg of Lumogen-loaded nanoparticles were inoculated in 0.5 g of porcine intestinal mucus and incubated for 2 h at 37 °C in order to ensure effective particle distribution after inoculation. Then, samples were observed in an epifluorescence microscope and videos of 10 s were recorded and imported to Fiji Image Software in order to convert the movement of each particle into individual trajectories within the mucus matrix. At least 100 individual trajectories were studied, and the experiment was replicated a further two times for each particle type. In order to compare the effective diffusion of each type of particle (Deff), after accounting the nanoparticle size results were expressed as % diffusion coefficient estimated as Deff/D°, where D° is the Stokes–Einstein equation for the diffusion of spherical particles through a liquid with low Reynolds number.

### 3.4. Animal Studies

All the animals were treated in accordance with institutional guidelines for the treatment of animals (Ethical Comity for the Animal Experimentation, of the University of Navarra, ref. 163–14).

### 3.5. Immunization of BALB/c Mice.

Eight week old BALB/c mice (20 ± 1 g) were randomized in groups of six animals and immunized orally. A single dose of phosphate-buffered saline (PBS), OMV-loaded nanoparticles (100 µg of extract) or free OMVs (100 µg of extract) were administered. F4 and F18 formulations were mixed at a 1:1 ratio; each concentration of the mixture was prepared individually. Blood and fecal samples were collected before immunization (Week 0) and weekly until four weeks post-immunization. Specific antibodies in serum (IgG1, IgG2a, IgA) and fecal samples (IgA) were determined by indirect ELISA at Week 0, 1, 2, 3 and 4 post-immunization.

Briefly, microplate wells (Immuno-Maxisorp, Nunc^®®^, Roskilde, Denmark) were coated with 10 µg/well of OMVs from ETEC (F4 or F18) diluted in coating buffer (60 mM carbonate buffer, pH 9.6), and incubated overnight at 4 °C. The plates were blocked with PBS containing 3% bovine serum albumin (BSA) for 1 h at 37 °C. Sample feces were treated with a protease inhibitor cocktail (Invitrogen, Carlsbad, CA, USA) and kept in PBS 3% milk at -20 °C until use. Serum or feces samples from mice were diluted in 1:80 PBS with 1% BSA in triplicates (1 h, 37 °C). After five washes with PBS-Tween20 buffer (PBS-T), the alkaline phosphatase-conjugated detection antibody, class-specific goat anti-mouse IgG/IgG2a/IgA/ (Sigma), was added for 1 h at 37 °C. The detection reaction was carried out by incubating the sample with H2O2-ABTS substrate–chromogen solution for 20 min at 37 °C. Absorbance was measured with an ELISA reader (Sunrise remote; Tecan-Austria, Austria) at a wavelength of 405 nm.

In order to determine the pattern of cyotkines (IL-2, IL-4, IL-6, TNFα, IFNγ, IL-17a, IL-10 and IL-22) elicited after immunization, naive and immunized mice were sacrificed by cervical dislocation at day 28 after immunization and their spleens were removed and placed in RPMI 1640 medium (Gibco-BRL, UK). The cellular suspensions were centrifuged at 380× *g* for 10 min, washed twice with PBS, and the splenocytes treated with lysis buffer (NH4Cl 0.15 M, KHCO3 10 mM, EDTA 14 0.1 mM) for 2 min to eliminate erythrocytes. The dispersions were centrifuged again (380 g, 5 min) and the resulting pellet were dispersed in RPMI 1640 medium supplemented with 1 IU/mL penicillin, 1 µg/mL streptomycin and 10% fetal bovine serum (Gibco-BRL, UK). The lymphocyte suspension was added to 96-well round-bottom microtitre plates (Iwaki, UK) (4 × 105 cells/well) and received one of the following different stimuli, F4-OMV (10 µg/mL) or F18-OMV (10 µg/mL), in a final volume of 200 µL per well. Negative control (PBS) and positive control (100 ng/mL + 4 µg/mL of PMA/Ionomicine used as mitogen) were used. The culture supernatants were collected for cytokine assay at 72 h after stimulation and were kept frozen at -80 °C. Cytokines were quantified by cytometry (Acoustic Focusing Cytometer Attune^®®^) using the Bead Array Th1/ Th2/ Th17 CBA (BD, USA).

### 3.6. Immunization of Pregnant Sows

Sows were divided into four groups. The first group, NPI (*n* = 6), orally received a single dose of 50 mg OMVs (25 mg OMV-F4 and 25 mg OMV-F18) encapsulated in zein nanoparticles coated with Gantrez–Manosamine. F4 and F18 formulations were mixed at a 1:1 ratio; each concentration of the mixture was prepared individually. Five weeks after primary immunization, the sows received a second immunization with the same amount of antigen.

The second group, NPII (*n* = 6), received a double dose of OMVs entrapped in nanoparticles (100 mg OMVs. Five weeks after the primary immunization, this group received a second immunization.

The third group of sows (*n* = 6) received one immunization with the commercial vaccine Suiseng^®®^. The remaining six pigs were used as a control and only received PBS orally. The born piglets were stabled with their mother until weaning.

Blood and fecal samples from sows were taken from the jugular vein at Weeks 0, 5, 7 and 8. Harvested sera were incubated at 56 °C for 30 min to inactivate the complement, and subsequently treated with kaolin (Sigma) to decrease the background reading in ELISA. The colostrum samples were taken on the day of birth and the blood samples of piglets were taken from the jugular vein seven days after the birth. Specific antibodies anti-OMVs were determined by indirect ELISA, as described above.

### 3.7. Statistical Analysis

All statistical significance analyses were carried out using the parametric one-way ANOVA test (with Tukey post hoc test). *p* values of < 0.05 were considered as statistically significant. All calculations were performed using SPSS^®®^ statistical software program (SPSS^®^ 15.0, *SPSS* Inc., Chicago, IL, USA).

## 4. Results

### 4.1. Characterization of OMVs-Containing Nanoparticles

Table 1 summarizes the main physico-chemical properties of the nanoparticles employed in this study. The nanoencapsulation of either OMV-F4 (F4-GM-NPZ) or OMV-F18 (F18-GM-NPZ) yielded homogenous batches of nanoparticles with a mean size close to 235 nm, similar to the empty ones. These nanoparticles displayed a negative zeta potential of about −32 mM, slightly higher that the values observed for the empty ones (about −37 mV). Interestingly, the payload of the resulting nanoparticles was calculated to be 60 µg OMV per mg nanoparticle.

OMV-loaded nanoparticles displayed a high capability to diffuse in intestinal pig mucus. Table 2 shows the diffusion coefficient in the intestinal mucus (Deff) of nanoparticles, measured by Multiple Particle Tracking (MPT), and the ratio as a percentage of these two parameters (Deff/D°). This last parameter was employed to compare the diffusion of the nanoparticles in intestinal pig mucus after normalising the effect of particle size. The capability of nanoparticles containing OMVs to diffuse in the intestinal mucus was found to be between 20 and 30 times higher than for empty nanoparticles.

### 4.2. Evaluation of the Immunogenicity in Mice

**Figure 1:**
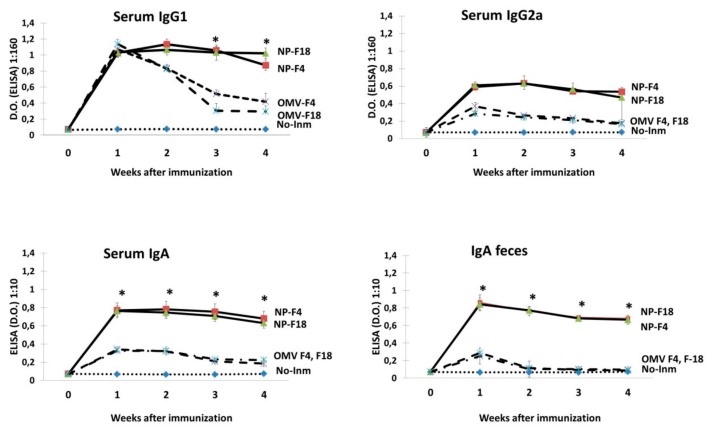
OMV-specific antibodies in immunized mice. The immunization was performed orally in BALB/c mice at Day 0 with either free OMVs (*Escherichia coli* F4 or F18 strains) or OMV encapsulated into nanoparticles (NP-F4, NP-F18). The ELISA values correspond with a 1:160 dilution of sera (**A**) and 1:10 dilution of fecal samples (**B**). Data are expressed as the mean OD405 nm ± SD at the indicated dilutions. *, *p* < 0.05 for immunized mice vs. non immunized control group).

Groups of six BALB/c mice were orally immunized with a single dose of OMVs (0.1 mg/mouse) from the F4 or F18 *E. coli* strains, either free or encapsulated into nanoparticles. A control group of non-immunized mice was also included. The elicited serum (IgG1, IgG2a and IgA) and fecal IgA specific antibodies were determined by indirect ELISA at Days 0, 7, 14, 21 and 28 post-immunization (Figure 1). High levels of IgG2a (Th1 response) and IgG1 (Th2 response) isotypes were detected. F4-GM-NPZ and F18-GM-NPZ presented a similar profile along the experiment, with levels significantly higher than those elicited by the non-encapsulated antigens. The adjuvant effect of nanoencapsulation was also significant with respect to the specific IgA in serum as well as feces (Figure 1).

**Figure 2:**
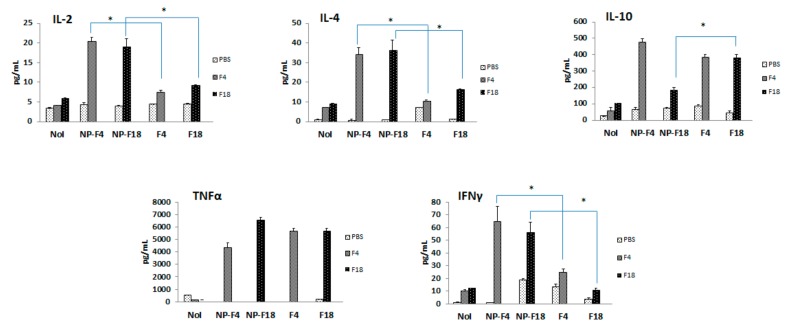
Splenic cytokine profile in immunized mice. BALB/c mice were orally immunized with one single dose of either free OMVs *Escherichia coli* F4 (F4), free OMVs *E. coli* F18 (F18) or OMVs encapsulated into nanoparticles (NP-F4, NP-F18) (*n* = 6). At Day 28 post-immunization, mice were sacrificed and spleen cells stimulated in vitro with PBS (light grey columns), F4 OMVs (grey columns) or F18 OMVs (black columns). Control non-immunized mice (NoI) were also included. The graphs show the levels of released cytokines from the primed splenocytes. *p* values of < 0.05 between OMVs encapsulated into nanoparticles and homologous free OMVs were considered as statistically significant (*).

The levels of different cytokines (IL-2, IL-4, IL-6, TNFα, IFNγ, IL-10, IL-17a and IL-22) were determined in the splenocytes of each group of mice 28 days after immunization (Figure 2). The encapsulation of OMVs in nanoparticles induced an increase in IL-2, IL-4 and IFN-γ with respect to the administration of free antigens.

### 4.3. Evaluation of the Immunogenicity Acquired after Oral OMVs Administration in Sows.

**Figure 3:**
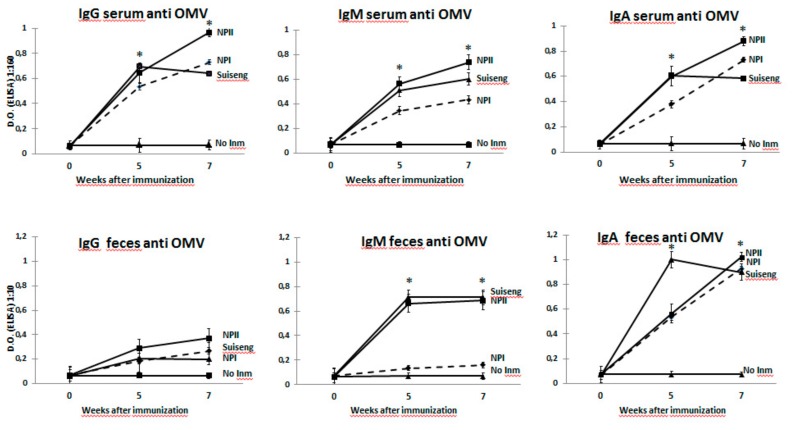
OMV-specific antibodies in immunized sows. Groups of eight week pregnant sows were immunized orally with the nanoparticle-based vaccine, and compared with a commercial vaccine (Suiseng^®^) that was administered intramuscularly. Nanoparticles contained OMVs from F4 and F18 strains. Two different doses were used: NPI (50 mg OMVs as a mixture between F4-OMV and F18-OMV 1:1) and NPII (100 mg OMVs as a mixture between F4-OMV and F18-OMV 1:1). The first dose of nanoparticles was administered to the sows between the 7th and 8th week of gestation. The second dose was administered between the 13th and 14th week of gestation. A control group of non-immunized sows was also included. The evolution of the elicited serum- (IgG, IgA and IgM) and fecal- (IgG, IgA and IgM) specific antibodies against OMV was determined by indirect ELISA 0, 5 and 7 weeks after the first immunization. Serum OMV-specific antibody profile diluted 1:160 (IgG, IgA or IgM), and fecal OMV-specific IgA profile diluted 1:10 (IgG, IgA or IgM) were quantified on weeks 0, 5 and 7 post immunization. Data are expressed as the mean OD405 nm ± SD at the indicated dilutions. *p* values of <0.05 between intervention and control groups were considered as statistically significant (*).

The serum antibody response induced by the experimental vaccines was assessed by ELISA. Five weeks after the first immunization, analysis revealed high levels of specific IgG in serum and IgA in feces. The higher specific antibody levels were found in the NPII vaccinated group. NPI and Suiseng^®®^ groups showed lower and similar levels between them. Regarding the response in mucosa, two weeks after the second immunization, the IgA levels decreased in animals receiving the commercial vaccine, while sows administered with the encapsulated vaccines reached the highest levels. In addition, three weeks after receiving the second immunization, IgG and IgA antibodies were also studied in colostrum collected on the day of labor, indicating that the NPII vaccine was the most effective in inducing specific antibodies. Significant differences were detected between NPI and NPII vaccines in eliciting specific IgG and IgM levels in serum. IgM levels in feces were also significant when compared to NPI and NPII (Figure 3).

**Figure 4:**
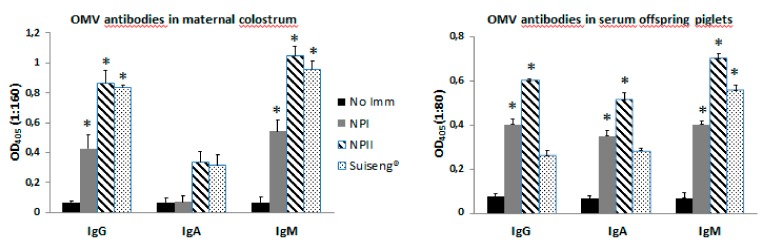
OMV-specific antibodies in colostrum and offspring piglets of immunized sows. Groups of eight-week pregnant sows were immunized orally with the nanoparticle based vaccine, and compared with a commercial vaccine (Suiseng^®^) that was administered intramuscularly. Nanoparticles contained OMVs from F4 and F18 strains. Two different doses were used: NPI (50 mg OMV) and NPII (100 mg OMV). The first dose of nanoparticles was administered to the sows between the 7th and 8th week of gestation. The second dose was administered between the 13th and 14th week of gestation. A control group of non-immunized sows were also included. OMV-specific antibody profile (IgG, IgA or IgM) was measured the day of labor in colostrum of sows. A control group of non-immunized sows was also included. An OMV-specific antibody profile (IgG, IgA or IgM) was also measured one week after labor in serum of piglets. Data are expressed as the mean OD405 nm ± SD at the indicated dilutions. *p* values of <0.05 between intervention and control groups were considered as statistically significant (*).

In maternal colostrum, there were significant differences between the non-immunized group and the immunized groups with NPI, NPII or Suiseng with respect to the specific IgG and IgM antibodies (Figure 4). The offspring piglets from immunized sows with NPI or NPII presented significantly higher levels of IgM, IgA and IgG antibodies than the ones from non-immunized mothers (Figure 4).

## 5. Discussion

Newborn and weaned piglets present an immature mucosal immune system, and consequently depend on the passive acquisition of maternal immunity via colostrum in order to cope with ETEC infections. Several maternal vaccines are on the market with this goal. These vaccines, which are mainly applied parenterally, contain different virulence factors such as fimbriae, LPS, outer membrane proteins (OMPs) and LT enterotoxins [24,25,26]. However, they tend to stimulate the systemic rather than the mucosal immune system required to provide protection against ETEC bacteria in the gut [17]. Oral immunization might overcome that problem but faces several barriers [27]. First, oral vaccines have to be able to successfully reach the intestine after diverse physico-chemical challenges, such as the extreme acidic pH in the stomach, the peristaltic gut movements and the antibacterial proteins and digestive enzymes that can degrade the antigens. Furthermore, an additional problem that oral vaccines have to face is the tolerogenic tendency of the mucosal tissues [28]. It is therefore critical that new vaccine candidates contain the right antigenic complex, but also the right adjuvant, to overcome those challenges. Several authors have evaluated the adjuvant effect of nanoparticles for oral administration [29,30,31,32,33]. Microencapsulated [34] and enteric-coated pellets [35] of ETEC fimbriae have been used to orally immunize pigs, however, these approaches did not result in a significant serum antibody response or in a reduction in colonization after infection. Vandamme et al. studied the oral adjuvant effect of Gantrez^®®^AN nanoparticles encapsulating F4 fimbriae in weaned pigs. However, they did not obtain complete protection against F4 ETEC, and an F4-specific serum antibody response could not be observed [36]. On the other hand, Gantrez AN nanoparticles are mucoadhesive and, due to the reactivity of the anhydride groups, covalent binding with antigens may occur in the aqueous environment. As a consequence, a fraction of the loaded antigen may be inactivated. In order to minimize these drawbacks, in this work OMVs were encapsulated into zein nanoparticles coated with a Gantrez–mannosamine conjugate (OMV-GM-NPZ). Zein nanoparticles can accommodate proteins without causing inactivation of the payload [37]. On the other hand, the employment of the Gantrez–mannosamine conjugate offers both mucus-permeating properties (due to its hydrophilic nature) and immunostimulatory features [21].

Mice studies revealed that OMV-GM-NPZ induce specific IgG1, IgG2a and IgA antibodies after a single oral dose. In contrast, the antibody levels elicited by non-encapsulated OMVs decreased earlier after immunization. The cytokine profile elicited in the animals immunized with encapsulated OMVs confirmed the immunoadjuvant properties of these particles. These differences are likely related to the protective and synergic effects of nanoparticles. First, it has been reported that zein-based nanoparticles conferred resistance to digestive enzymes [38]. Second, Gantrez–mannosamine has demonstrated immunostimulant properties [20,29,30]. Third, the encapsulation of antigens creates high-density antigen surfaces that increases the possibility of antigen recognition and/or capture by the antigen presenting cells [31].

These results support the use of zein–Gantrez–mannosamine nanoparticles for oral vaccination. Given that the long-term goal of our research is to develop a vaccine to protect piglets from infection, an additional study was performed in pregnant sows. As indicated before, newborn and weaned animals are extremely susceptible to ETEC infections due to the lack of protection at birth. During this time, resistance to infection depends mainly on the actions of the innate defense mechanisms and specific antibodies transferred passively from sow to piglet through the colostrum and milk. This maternally-derived immunity must provide sufficient protection during the period in which the piglet gradually develops its own active immunity. For this purpose, we studied whether this new vaccine is able to induce an appropriate immune response, capable of being transferred to the piglets through colostrum. Results show that the immunization with OMVs containing nanoparticles elicited specific antibodies and, more importantly, the piglets whose mothers were vaccinated with this new vaccine presented high levels of antibodies. Several maternal vaccines are on the market [24]. These are mainly applied parenterally in the pregnant sow; passive protection thus decreases rapidly and, consequently, the newly weaned piglet becomes highly susceptible [8].Therefore, the results obtained with OMVs encapsulated in the here-described zein nanoparticles support further studies into the immunization of sows to follow the protective effect after experimental challenge of the offspring piglets. However, the projection of any formulation to be applied in the pregnant target is not straightforward. Large animal trials are needed to determine the protective efficacy of these new maternal vaccine approaches.

## 6. Conclusions

Enterotoxigenic *Escherichia coli* (ETEC) infections produce significant economic losses in swine husbandry. Given the promising evidence of nanotechnology in vaccination, we present here an affordable and easy-to-produce nanoparticle vaccine candidate against ETEC, based on zein and the immunomodulatory polymer Gantrez^®®^ AN119 (poly-methyl vinyl ether-co-maleic anhydride). Results show the potential of ETEC outer membrane vesicles loaded into these mucopenetrating nanoparticles for oral vaccination in pregnant sows. The presence of antibodies in offspring piglets indicates that this vaccination strategy has real potential with respect to the transfer of maternal antibodies to the offspring.

## Figures and Tables

**Table 1:** Physico-chemical characterization of outer membrane vesicles (OMV)-containing nanoparticles. GM-NPZ: Empty zein nanoparticles coated with Gantrez-mannosamine conjugate; F4-GM-NPZ: Outer membrane vesicles from F4 *Escherichia coli* encapsulated in GM-NPZ; F18-GM-NPZ: Outer membrane vesicles from F18 *Escherichia coli* encapsulated in GM-NPZ. Data expressed as mean ± SD (*n* = 3).

Formulation	Size (nm)	Polydispersity Index	Zeta Potential (mV)	Yield (%)	µg OMV/ mg NP
**GM-NPZ**	227 ± 6	0.108 ± 0.04	−37.1 ± 0.8	88 ± 2	n.a.
**F4-GM-NPZ**	240 ± 3	0.151 ± 0.03	−32.5 ± 1.8	82 ± 5	60 ± 1.5
**F18-GM-NPZ**	231 ± 4	0.132 ± 0.03	−31.9 ± 0.8	81 ± 3	60 ± 1.5

**Table 2:** Diffusion behaviour of the different formulations tested in intestinal pig mucus. Data expressed as mean ± SD (*n* = 3).

Formulation ^a^	Water Diffusion (D°) ^b^ cm^2^·S^−1^ ×10^−9^	Mucus Diffusion (Deff) ^c^ cm^2^·S^−1^ × 10^−9^	% Diffusion Coefficient (Deff/ D°) ^d^	R ^e^
GM-NPZ	17.23	0.0072 (±0.0062)	0.0419	1
F4-GM-NPZ	18.62	0.2449 (±0.0011)	1.3156	31.4
F18-GM-NPZ	19.42	0.1835 (±0.0054)	0.9455	22.6

^a^ GM-NPZ: Empty zein nanoparticles coated with Gantrez–mannosamine conjugate; F4-GM-NPZ: Outer membrane vesicles from F4 Escherichia coli are encapsulated in GM-NPZ; F18-GM-NPZ: Outer membrane vesicles from F18 Escherichia coli are encapsulated in GM-NPZ. ^b^ D: diffusion coefficient in water according to Stokes–Einstein equation. ^c^ Deff: diffusion coefficient in mucus. ^d^ % Diffusion coefficient: relative efficiency of particles diffusion estimated as Deff/ D° ratio. ^e^ R: ratio of % diffusion (Deff/ D°) of the formulations tested in comparison with GM-NPZ.
